# Cooperative predation in the social amoebae *Dictyostelium discoideum*

**DOI:** 10.1371/journal.pone.0209438

**Published:** 2019-01-09

**Authors:** Michelle Rubin, Amber D. Miller, Mariko Katoh-Kurasawa, Christopher Dinh, Adam Kuspa, Gad Shaulsky

**Affiliations:** 1 Graduate Program in Integrative Molecular and Biomedical Sciences, Baylor College of Medicine, Houston, TX, United States of America; 2 Department of Molecular and Human Genetics, Baylor College of Medicine, Houston, TX, United States of America; 3 Verna and Marrs McLean Department of Biochemistry and Molecular Biology, Baylor College of Medicine, Houston, TX, United States of America; Université de Genève, SWITZERLAND

## Abstract

The eukaryotic amoeba *Dictyostelium discoideum* is commonly used to study sociality. The amoebae cooperate during development, exhibiting altruism, cheating, and kin-discrimination, but growth while preying on bacteria has been considered asocial. Here we show that *Dictyostelium* are cooperative predators. Using mutants that grow poorly on Gram-negative bacteria but grow well on Gram-positive bacteria, we show that growth depends on cell-density and on prey type. We also found synergy, by showing that pairwise mixes of different mutants grow well on live Gram-negative bacteria. Moreover, wild-type amoebae produce diffusible factors that facilitate mutant growth and some mutants exploit the wild type in mixed cultures. Finding cooperative predation in *D*. *discoideum* should facilitate studies of this fascinating phenomenon, which has not been amenable to genetic analysis before.

## Introduction

Cooperative predation is pervasive across phylogeny [1]. Like many other social behaviors, it exhibits three central characteristics–correlation between organism density and fitness, production of common goods, and cheating [2]. An example of the relationship between predator density and fitness is seen in wolves, in which large packs can hunt large prey such as bison, whereas smaller packs hunt smaller prey such as elk [3]. The production of common goods is exemplified in *Myxococcus xanthus*, which are predatory bacteria that kill their prey (other bacteria) by secreting lytic enzymes into the shared extracellular milieu [4]. Cheating is observed when individuals exploit the benefits of cooperative predation without contributing their fair share [5]. For example, in Taï chimpanzees, cheaters do not participate in the energy-intensive hunt, but they benefit from the meat obtained by the hunters [6].

*D*. *discoideum* is a genetically tractable model system for the study of cooperation at the cellular level. The amoebae prey on bacteria by phagocytosis and propagate as unicellular organisms [7]. Upon starvation, *D*. *discoideum* undergoes aggregative development that exhibits many characteristics of sociality and cooperation [8], including altruism and cheating [9, 10]. Sociality in *Dictyostelium* development is mainly considered in the context of cell-type differentiation. The starving amoebae begin their development by aggregation, so the multicellular structures often contain different genotypes. As the cells differentiate, some of the cells make an altruistic sacrifice and become inviable stalk cells, while the others become viable spores. Social phenomena such as cheating [10], counter-cheating [11], and allorecognition [12] have become central components in the investigation of sociality in a variety of contexts and established *Dictyostelium* as a choice model system for the investigation of sociality [2, 8, 13–17]. Although the developmental stage is social, the growth stage has been largely considered asocial [14]. One report, however, demonstrated a density effect during growth on certain bacterial strains, suggesting sociality during growth [18]. That observation, and the genetic complexity that underlies the ability of the amoebae to distinguish between bacterial food sources [19], prompted us to test whether *D*. *discoideum* were indeed social predators.

Predation is the consumption of one living organism, or some it its parts, by another living organism for food. The term predation may have various meanings in different contexts, so here we define the amoeba *D*. *discoideum* as the predator and bacterial cells of several species as the prey. The amoeba consumes its bacterial prey by phagocytosis in a cell-autonomous manner. The prey is not defenseless, and the amoebae employ several intracellular mechanisms to kill their prey after internalization, including vesicle acidification, lytic enzymes, reactive oxygen species, and metal ions [20]. Other aspects of predation in this system include prey-tracking by chemotaxis of the amoebae toward bacteria [21], and extracellular killing of bacteria by the amoebae [22]. The observation of extracellular signals and processes indicate that non-cell autonomous factors might also be involved in this system, suggesting that predation could also be cooperative.

The definition of social predation is rather intricate and somewhat context-dependent [1]. One essential part of the definition is the co-occurrence in time and space of two or more predators and their prey. True social predators must also gain an advantage from the cooperation, mainly due to the cost of sharing the food. Moreover, almost every social predator can prey as a solitary organism as well, so cooperative predation is often facultative. Finally, social predation is a dynamic process that balances the costs of competition with the benefits of cooperation, so its effects on fitness are conditional [1]. In the amoeba-bacteria interaction, the degree of co-occurrence can be determined experimentally by combining the two species at various proportions. Here we evaluate the cost and benefit to the predator by measuring the growth of the amoebae at different cell densities with an initial excess of prey bacteria. Very low amoebae cell density is used to favor low co-occurrence (and thus solitary predation) and higher amoebae cell density is used to favor co-occurrence. These results, along with our analyses of mutant amoebae, suggest that *Dictyostelium* is a social predator.

## Materials and methods

### Strains and mutation screens

All the strains were derived from the laboratory wild-type *D*. *discoideum* strain AX4 [23]. The relevant genotypes are indicated in S1 Table. We generated mutant stains MR01–08 by insertional mutagenesis of a large population of AX4 cells [24] and screening for poor growth on the Gram(–) bacteria *Klebsiella pneumoniae* and robust growth on the Gram(+) bacteria *Bacillus subtilis* as described [19] with the exception that the amoebae were incubated in association with dilute bacteria in small spots on SM-agar plates such that the two species grew together before the growth phenotype was determined. We generated a new *cadA*-null allele by homologous recombination using the pCR2.1-TOPO plasmid [25]. To fluorescently label the amoebae, we transformed them with either pDEX H-RFP or pDEX H-GFP [26, 27]. We grew all *D*. *discoideum* strains axenically in HL-5 liquid medium [28] supplemented with 4 μg/mL blasticidin S as needed. We grew the fluorescently labelled strains with 10 μg/mL G418.

### Bacterial cultures

We grew the Gram(–) bacteria *K*. *pneumoniae* and the G(+) bacteria *Staphylococcus aureus* in nutrient medium [19] to OD_600_ of 5.0–6.0, collected the cells by centrifugation and resuspended in an equal volume of KK2 buffer (20 mM KH_2_PO_4_/K_2_HPO_4_, pH 6.8). We heat-killed the bacteria by autoclaving a bacterial suspension at 121°C for 45 minutes.

### Submerged culture

We incubated amoebae with heat-killed *K*. *pneumoniae* or *S*. *aureus* in KK2 buffer in submerged culture. After 16 hours, we washed the cells once with KK2 buffer, added the amoebae to the respective live bacteria resuspended in 5ml KK2 buffer, placed them in 60mm dishes and incubated at 22°C without shaking or agitation. At each time point we resuspended the cells by repeated pipetting and counted them on a hemocytometer. Each time point, condition, and replicate, were done in a separate plate and the cultures were discarded after counting. We counted three technical replicates for each time point and repeated each assay at least three times.

### Allee effect analysis

We calculated the density fold-change starting at the four-hour time point until the 26-hour time point for AX4 and MR08. At each time point, we determined the fold-change ratio by dividing the cell density at that time point by the cell density at the previous time point. The fold-change ratio is directly proportional to the population fitness. We performed this calculation on each technical replicate and then averaged the replicates at each time point. We also calculated the average cell density at each time point. We plotted the average fold-change against the average density. In non-cooperative predators, one expects a downward trend in this graph, because increasing predator density increases the competition between the predators and leads to overall loss of fitness. An upward trend at any point in the graph suggests cooperativity [29, 30].

### Synergy

We mixed mutant strains at equal proportions, grew them with *K*. *pneumoniae* and counted them as above. We also grew the wild type as well as each mutant strain in pure populations at the same time. We plotted the cell density as a function of time and calculated the area under the curve (AUC) for each strain and strain mix for the first 12 hours. We subtracted the AUC of the mutant mix from the AUC of the wild type, computed a z-score for the differences and plotted the data as a heat map. The raw data are provided in S2 Table. In all cases, a positive z-score indicates synergy. We calculated statistical significance by one-way ANOVA and post-hoc Tukey’s HSD test for pair-wise comparisons at each time-point, comparing the mixed population with the respective pure mutant populations. In general, we considered mixes that displayed *p* ≤ 0.05, compared to pure mutant populations, as synergizing and mixes that displayed *p >* 0.05 as non-synergizing.

### Testing soluble cooperation factors

We used six-well Greiner Bio-One ThinCert Cell Culture Inserts with 0.4 μm pore polyester membrane to physically separate distinct cell populations while allowing diffusion of soluble molecules. We incubated the six-well dish in a humid chamber at 22°C.

### Cheating

We incubated unlabeled wild-type cells and fluorescently-labelled mutant cells in pure populations (5x10^4^ cells/mL) and as mixed populations of equal proportions (2.5x10^4^ cells/mL each) with pre-grown Gram(–) *K*. *pneumoniae* bacteria. At 0 and 8 hours, we collected and washed the cells twice with 20mM EDTA made in KK2 buffer and then determined the ratio of unlabeled to labeled cells by cell counting under fluorescence microscopy. At 0 hours, we counted between 250–350 cells and at 8 hours we counted between 500–600 cells.

### Statistical analysis

We performed statistics analyses with the R Project for Statistical Computing and the R-commander package [31–34]. The statistical tests are indicated in each figure legend.

### REMI insertion site analysis

We prepared genomic DNA and performed whole-genome sequencing of the *D*. *discoideum* strains as described [35]. We aligned the sequences to the reference AX4 genome [36] and then to the pBSR1 sequence [37] using the Integrative Genomics Viewer software [38, 39]. We verified the plasmid insertion sites by polymerase chain reaction (PCR) using primers specific to the BSR cassette and to the insertion site. The insertion sites are described in S1 Table.

## Results

To facilitate the study of cooperation between amoebae during growth on bacteria, we mutated *D*. *discoideum* cells and screened for mutant strains that grow poorly on Gram(–) bacteria but grow well on Gram(+) bacteria and on heat-killed Gram(–) bacteria (S1 Table).

### Growth on bacteria depends on cell density

**Fig 1:**
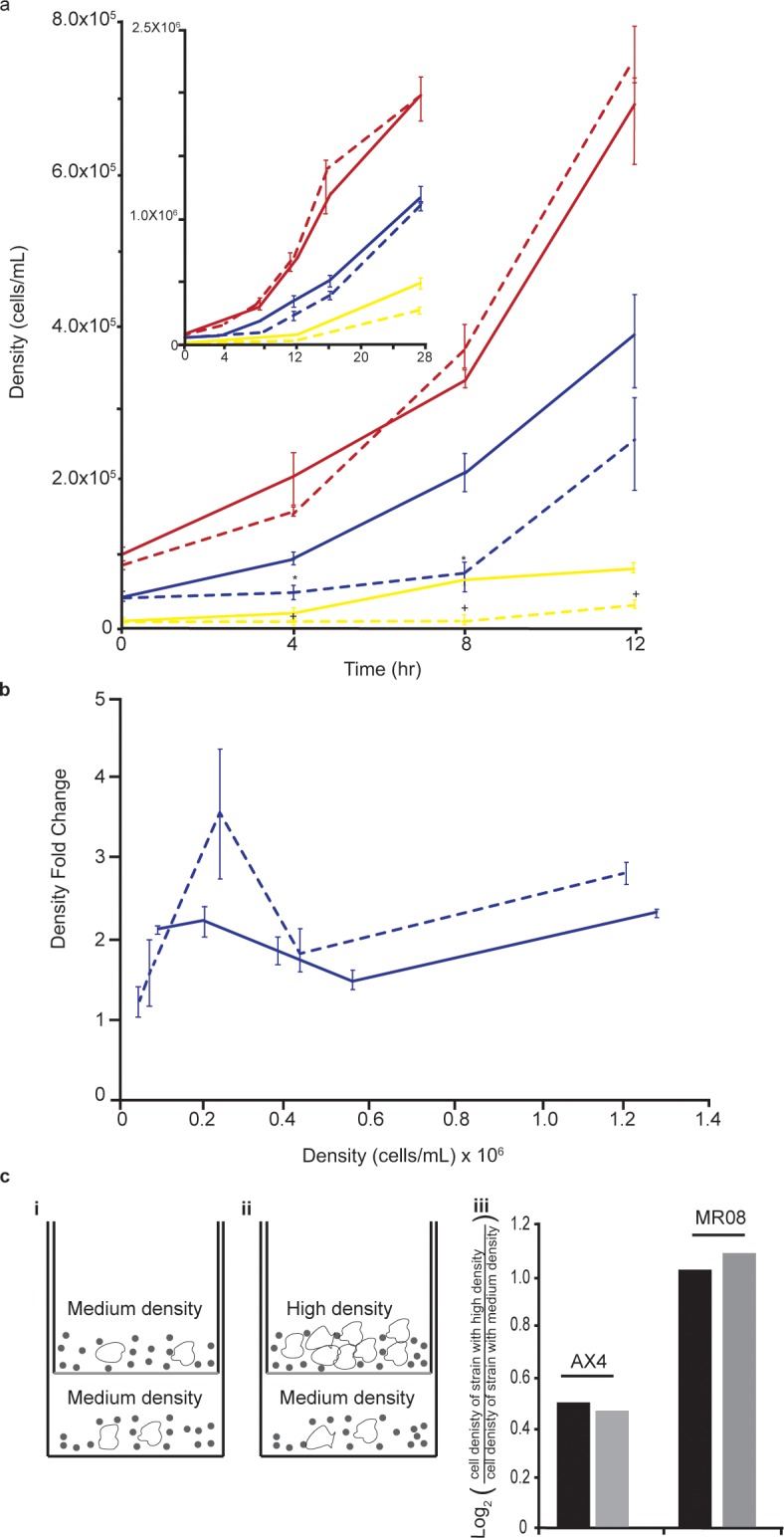
Cell density affects growth. (a) We placed *D*. *discoideum* cells in submerged cultures in association with *K*. *pneumoniae* at the following densities: yellow– 1x10^4^ cells/mL, blue– 5x10^4^ cells/mL, red– 1x10^5^ cells/mL. We incubate the cultures and counted the amoebae at the indicated times. The graph shows the calculated cell density (y-axis, cells/mL) as a function of time (x-axis, hours). Solid lines: wild type; dashed lines: MR08 mutant. Each data point represents the mean of three independent replications and the bars represent the standard error of the mean. The inset shows the full growth curve, including the 16hr and 26hr time points. * P ≤ 0.05 for 5x10^4^ cells/mL, + *P* ≤ 0.05 for 1x10^4^ cells/mL; independent sample t-test for pair-wise comparisons between the wild type and the mutant at each time-point. (b) Allee effect: we calculated the change in density from the 5x10^4^ cells/mL growth curve and plotted the fold change (y-axis) as a function of cell density (x-axis, cells/mL) for the wild type (solid line) and for the MR08 mutant (dashed line). (c) We placed pure populations of wild-type or MR08 cells at 5x10^4^ cells/mL (medium density) in association with *K*. *pneumoniae* at the bottom of six well culture dishes. The white shapes represent amoebae and the black circles represent bacteria (i, ii). We placed semi-permeable inserts into the wells and inoculated them with *K*. *pneumoniae* bacteria and with the respective amoebae strain at medium density (i, 5x10^4^ cells/mL) or high density (ii, 1x10^5^ cells/mL). We incubated the dishes and counted the cells in the bottom well (iii) after 4 hours (black bars) and after 8 hours (grey bars). We plotted (y-axis) the log_2_ of the ratio between the mean cell density in the presence of inserts containing amoebae at high density (ii) and the mean cell density in the presence of inserts containing amoebae at medium cell density (i). The means are from 3 independent replicates for each condition.

To test whether *Dictyostelium* cells display a positive correlation between the number of predators and their overall fitness, a phenomenon known as the Allee effect [30], we inoculated amoebae at different densities with excess prey (live bacteria) in submerged cultures and followed their growth for several generations. We tested wild-type AX4 amoebae as well as MR08 –a mutant strain that displays poor growth on Gram(–) bacteria. We first grew the amoebae on heat-killed bacteria, allowing them to reach exponential growth. We removed the dead bacteria, mixed the amoebae with live Gram(–) bacteria, and incubated them in submerged cultures in buffer, so that the bacteria would not grow. We collected samples at 4-hour intervals for the first 16 hours to examine the growth parameters, and another sample at 26 hours, to examine the long-term effects. We counted the number of amoebae, and calculated their density at each time point (Fig 1). At the highest inoculation density, 1x10^5^ cells/mL, the wild-type cell density doubled roughly every four hours (mean, 4.49h; range, 3.5–5.47), indicating exponential growth with almost no lag. We noted that growth was accelerated between 12 and 16 hours and then somewhat attenuated between 16 and 26 hours. The faster growth period could be the result of increased cell density, as the amoebae consume the bacteria and divide, suggesting a cooperative effect. The slower growth period could be due to increased competition–the culture conditions do not include shaking so as the amoebae consume the bacteria in their immediate area, they may face competition from their siblings for the remaining food. When the inoculation density was decreased to 5x10^4^ cells/mL, we observed slow growth for the first 4 hours, and the cell density increased more slowly throughout the experiment. In this case we also observed a general trend of faster growth as the cell density increased, but we did not observe slower growth between 16 and 26 hours, presumably because the amoeba cell density did not reach a point in which the cost of competition for food was greater than the benefit of increased density. The density-dependence trend was even more pronounced at 1x10^4^ cells/mL, with a rather long lag in the first 8 hours, faster growth afterwards, and no reduced growth between 16 and 26 hours (Fig 1A). The MR08 mutant cells grew as well as the wild type at the highest cell density of 1x10^5^, but exhibited a longer lag when incubated at 5x10^4^ cells/mL. When incubated at the lowest cell density of 1x10^4^ cells/mL, the mutant cells exhibited an even longer lag and slower growth (Fig 1A). Using the middle density (5x10^4^ cells/mL), we also plotted the fold change in population density, which is a proxy of fitness, as a function of population density for both strains (Fig 1B). Both graphs exhibited a peak around a density of 3x10^5^ cells/mL, with the mutant plot displaying a much sharper increase, suggesting an Allee effect [29]. These findings are also consistent with empirical evidence on the dependence of fitness on predator group size from a simulation model and from field observations in predators such as lions, wolves, birds and worms [1].

To further characterize the density-dependence effect, we tested whether cells at high density might produce a soluble common good that would stimulate the growth of cells at a lower density. We used cell culture inserts with semi-permeable membranes on the bottom to physically separate cell cultures at different densities from one another while the soluble media were shared (Fig 1C) [40]. We placed cells of each strain at two different densities together with Gram(–) bacteria in separate chambers. We used medium cell density (5x10^4^ cells/mL) in the bottom chamber (well) for both the control and the experiment. In the upper chamber (insert), we used medium cell density in the control (5x10^4^ cells/mL, Fig 1C, i) and high cell density in the experiment (1x10^5^ cells/mL, Fig 1C, ii). We incubated the cultures and counted the cell density in the well after 4 and 8 hours. We found that the less dense amoebae in the well benefited from sharing media with the more dense population both in the wild type and in the MR08 mutant (Fig 1C, iii). This finding further supports the density dependence hypothesis and suggests the involvement of a secreted common good.

We tested eight more mutant strains with compromised growth on Gram(–) bacteria and observed similar density dependence in most cases (S1 Fig). These findings indicate that *D*. *discoideum* amoebae display a positive correlation between density and growth on live bacteria, consistent with the hypothesis of cooperative predation. The results also suggest that the mutant strains are compromised in this cooperative property.

Although most of the strains exhibited fast growth at later times, the slow growth observed at early times would probably confer a selective disadvantage if the mutants were in direct competition with the wild type. Therefore, it is possible that cooperation has a role in *D*. *discoideum* growth, although it is hard to estimate the potential implications of this role in nature.

### Synergy between mutant strains

**Fig 2:**
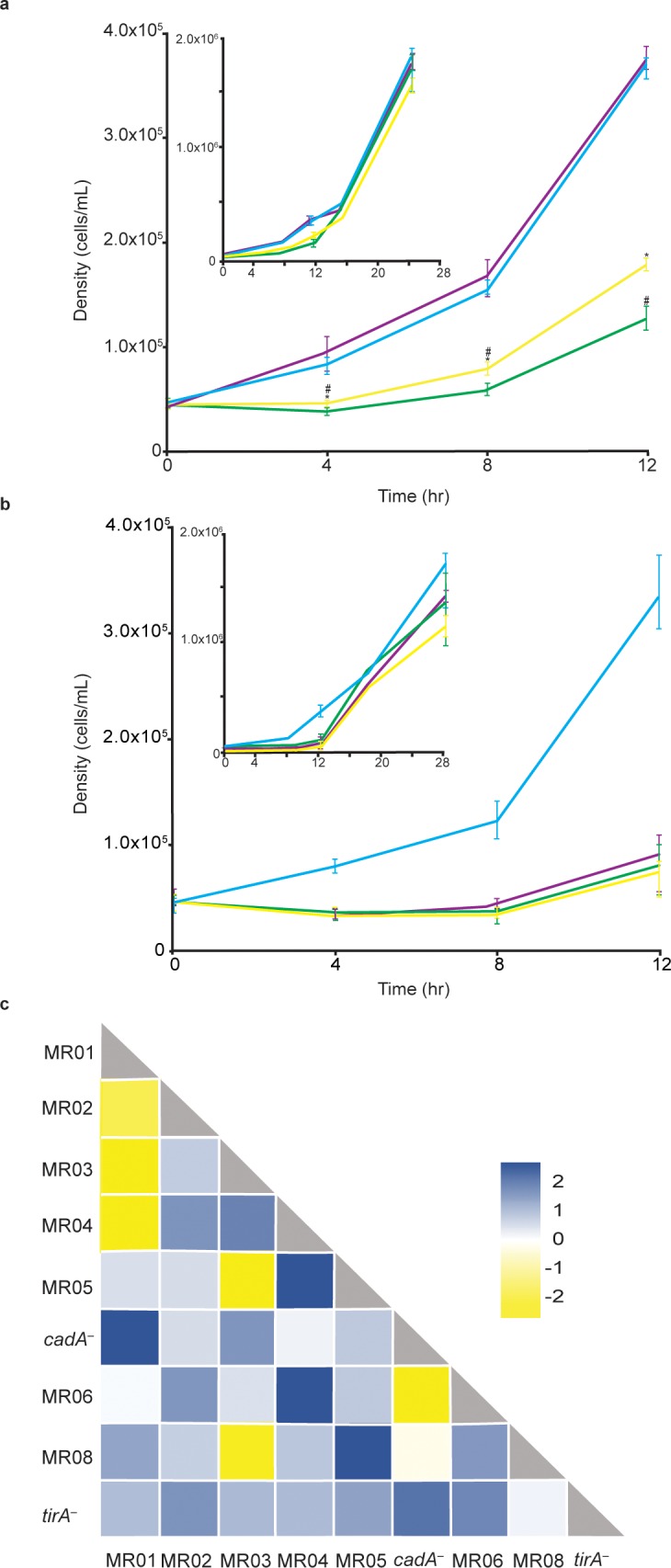
Synergy between *D*. *discoideum* mutants during growth. We incubated wild-type and mutant cells in pure populations and in mixes in submerged cultures in association with *K*. *pneumoniae* at a density of 5x10^4^ cells/mL and counted the cells at the indicated times. Each experiment was performed in three independent replicates; the results are reported as the means and the bars represent the standard error of the mean. (a) An example of synergy: cyan–a pure population of wild-type cells, green–a pure population of MR02 mutants, yellow–a pure population of MR04 mutants, purple–a 1:1 mix of MR02 and MR04. The inset shows the full growth curve including the 16hr and 26hr time points. * *P* ≤ 0.05 for MR02, # *P* ≤ 0.05 for MR04. (b) An example of non-synergy: cyan–a pure population of wild-type cells, green–a pure population of *cadA*^*–*^, yellow–a pure population of MR06, purple–a 1:1 mix of *cadA*^*−*^and MR06. The inset shows the full growth curve including the 16hr and 26hr time points. (c) We made all possible pairwise mixes of 9 mutant strains and recorded synergy (blue) and non-synergy (yellow). We calculated a z-score for each mix by determining the difference between the area under the curve between the mixed mutant population the wild-type. In all cases (a-c) we calculated statistical significance by one-way ANOVA and post-hoc Tukey’s HSD test for pair-wise comparisons at each time-point, comparing the mixed population with the respective pure mutant populations.

A more direct indicator of cooperation is a synergy test, in which two mutant strains are grown in mixed cultures. If the behavior of the mixed culture is different from the sum of the behaviors of the pure cultures, then the mutations are thought to compromise a cooperative process [41]. We tested this possibility by mixing the nine mutants in all possible pairwise combinations at the medium density (5x10^4^ cells/mL) and monitoring their growth on live Gram(–) bacteria (Fig 2). In the controls, the wild-type cell density doubled roughly every 4 hours and the pure mutant populations exhibited compromised growth for the first 12 hours. In some cases, the two mutants synergized and the mixed population doubled roughly every four hours, just like the wild type (Fig 2A). In other cases, the mutants did not synergize, and the mixed population grew as poorly as the pure populations, indicating that the synergy test is not indiscriminant and suggesting that the underlying mutations modified genes in common pathways (Fig 2B). Overall, we found that 34 of the 45 pairwise mixes demonstrated synergy (blue and white in Fig 2C) whereas 11 did not (yellow in Fig 2C). One of the strains, *tirA*^*–*^, synergized with all the other strains and none of the strains failed to synergize altogether. These results support the hypothesis that predation on live bacteria is a cooperative process and suggest that none of the mutations conferred a cell-autonomous phenotype. Moreover, the distinction between synergizing and non-synergizing groups of mutants suggests that cooperative predation involves multiple genetic pathways.

### Cooperative predation depends on prey type

Cooperative predation behavior may depend on the type of prey, as seen in wolves [3]. We tested the relationship between prey-type and predator density in *D*. *discoideum* by measuring growth of the mutant strains on heat-killed Gram(–) bacteria and on live Gram-positive [Gram(+)] bacteria. When mixed with heat-killed Gram(–) bacteria, the cell density of wild-type and mutant cells doubled roughly every four hours (S2 Fig). The cell density of wild-type amoebae incubated with Gram(+) bacteria also doubled every 4 hours as did eight of the nine mutants, confirming and extending the findings of the original genetic screen that yielded the mutants (S3 Fig). These findings indicate that the growth defect observed in the mutants is dependent on the type of prey, further suggesting that predation is indeed cooperative.

### Mutants are compromised in the production of soluble common goods

**Fig 3:**
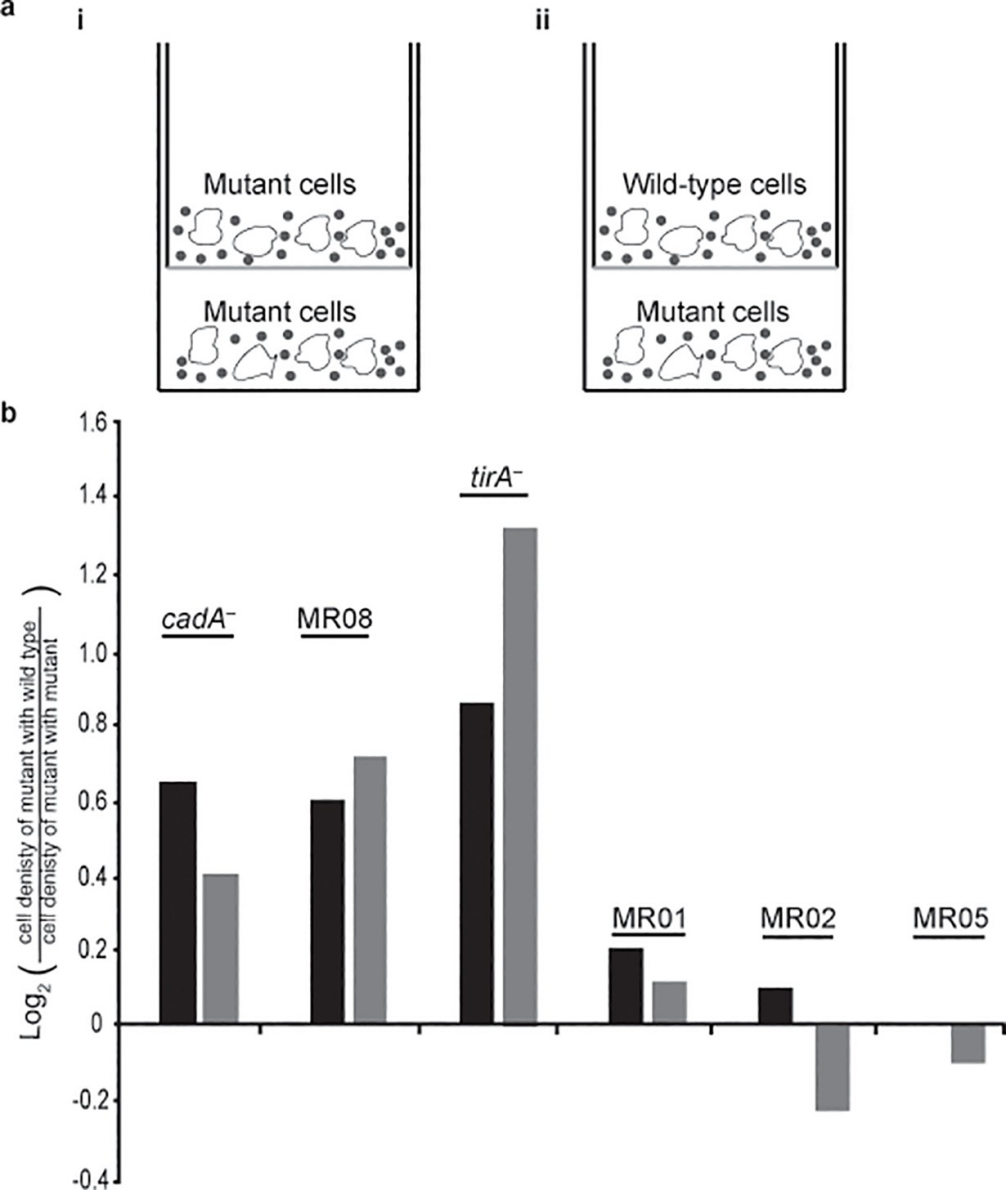
Cooperative growth is mediated by soluble factors. (a) We placed pure populations of wild-type and mutant cells at 2.5x10^4^ cells/mL in submerged cultures in association with *K*. *pneumoniae* at the bottoms of two cell culture wells. We placed inserts in these wells, in which submerged cultures of amoebae (white shapes) and bacteria (black circles) were deposited on a membrane–one insert with matching mutant amoebae (i) and one with wild-type amoebae (ii). (b) We incubated the cells for 4-hours (black bars) and 8-hours (grey bars) and counted the mutant cells in the bottom wells. For each strain, we plotted (y-axis) the log_2_ of the ratio between the mean mutant cell density in the presence of inserts containing wild-type cells (ii) and the mean mutant cell density in the presence of inserts containing mutant cells (i). We performed three independent replications for each experiment. A positive value indicates that the mutant grew more in the presence of wild-type cells. Strain names are indicated above the bars. Statistical significance is shown in S4 Fig.

Production of common goods is a characteristic feature of cooperation and our data already suggest that cooperative predation involves a secreted common good (Fig 1C). We hypothesized that the mutants might be compromised in the production of such common goods. To test the hypothesis, we used the cell culture insert system and tested whether wild-type amoebae could provide the mutants with secreted, stable common goods. In the control, we placed cultures of identical mutant amoebae in both chambers (Fig 3A, i). In the experiment, we placed wild-type amoebae in the insert and mutant amoebae in the well (Fig 3A, ii). In all cases, the amoebae were mixed with excess live Gram(–) bacteria. We counted the amoebae after 4 and 8 hours and calculated the cell density. We then calculated the ratio between the cell density of the mutant after co-incubation with the wild type and the cell density of the mutant after co-incubation with the same mutant. We found three mutants, *cadA*^*–*^, MR08, and *tirA*^*–*^, that benefited from co-incubation with wild-type amoebae as evidenced by a doubling of the cell density (Fig 3B), suggesting that a stable soluble common good was indeed secreted by the wild type and utilized by the mutant. We also found three mutants, MR01, MR02, and MR05, that did not exhibit increased cell density under these conditions (Fig 3B), indicating that the assay system is not indiscriminant. For completeness, we measured the changes in cell density in the well and in the insert for wild-type amoebae that were co-incubated with wild-type, mutant with mutant, and mutant with wild-type for all of the mutants tested and found internally consistent results (S4 Fig). It was formally possible that mutant growth was conferred by dead bacteria, or small fragments thereof, that passed through the semi-permeable barrier. We found no support for this possibility by microscopic examination of empty wells that were incubated with inserts that contained wild-type amoebae and bacteria. Overall, the results shown in Fig 3 suggest that *D*. *discoideum* secretes a stable, soluble common good that facilitates predation of live Gram(–) bacteria, further supporting the cooperative predation hypothesis.

### Cheating behavior in cooperative predation

**Fig 4:**
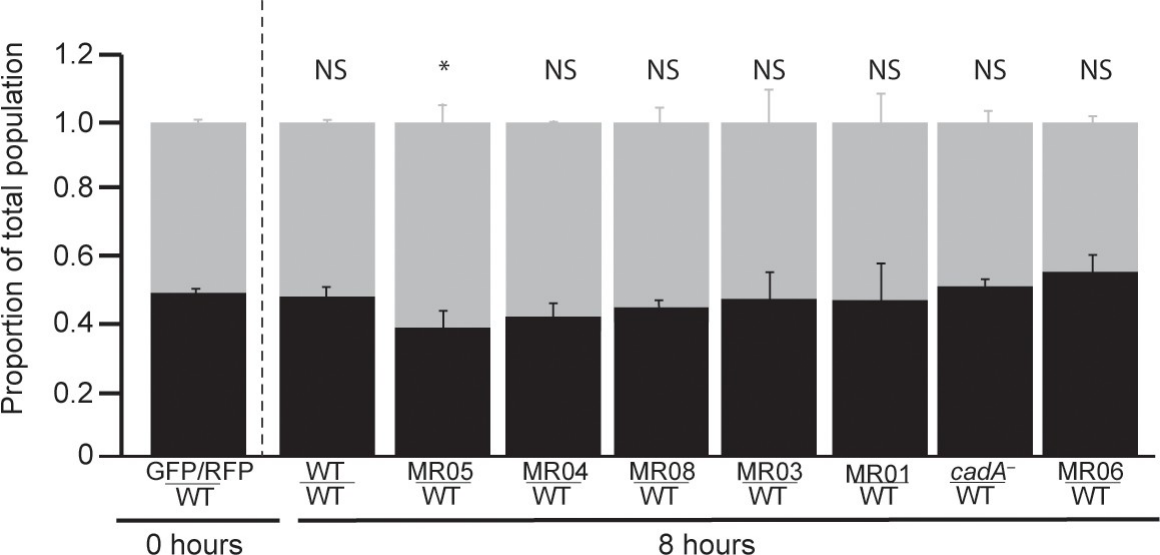
Cheating ability of mutants during growth. We incubated differentially labelled (RFP or GFP) mutant cells in 1:1 mixes with unlabeled wild-type amoebae at 5x10^4^ cells/mL in submerged cultures with *K*. *pneumoniae*. We counted the number of labeled and unlabeled cells by fluorescence microscopy immediately after plating (0 hours) and after 8 hours, and calculated the ratios between the labeled and unlabeled cells (y-axis). The bar graphs show the proportion of each strain as the mean of 3 independent replicates; error bars represent the standard error of the mean. Strain names are indicated on the x-axis (fluorescently-labelled cells on top, grey bars; unlabeled wild type on the bottom, black bars). MR01, MR03, MR04, MR05, and wild-type cells were tagged with GFP; MR08, *cadA*^–^, and MR06 were labeled with RFP. We tested each instance for difference from the 0-hours sample by a Student t-test and indicated the results above the bars: NS, not significant; * *P* ≤ 0.05.

Cooperation is often associated with cheating [5]. To test whether cheating occurs during cooperative predation, we tagged the mutant strains with either GFP or RFP and mixed them with unlabeled wild type. We performed eight mixes in which a mutant strain was tagged with GFP or RFP and mixed with unlabeled wild-type cells. We determined the ratio of fluorescently-tagged cells to unlabeled cells at the onset (0 hours) and after 8 hours of incubation (Fig 4). One of seven mutants, MR05, exhibited cheating, as the ratio of fluorescently-labeled mutant cells to unlabeled wild-type cells changed from 50:50 to 60:40. In the other instances, the proportions were indistinguishable from the original 50:50 ratio, suggesting that the assay is not indiscriminant. Interestingly, two strains that benefited from a possible secreted common good, *cadA*^*−*^and MR08, did not exhibit cheating in this assay. This finding suggests that the genes altered in these two mutants might be pleiotropic, as seen in cooperation genes that function during *Dictyostelium* development [42]. Overall, these findings point to the possibility that predation might involve cheating, as expected from a cooperative process.

## Discussion

Based on the data presented here we propose that *D*. *discoideum* is a cooperative predator. This conclusion is supported by the findings that *D*. *discoideum* amoebae exhibit density-dependent growth and by the observation that many of the mutants can synergize with one another. The diverse synergy behaviors also suggest that cooperative predation in *D*. *discoideum* utilizes multiple pathways. Most of the genes mutated in the strains we used do not have clearly defined functions and their annotations do not fall into obvious common functional groups (S1 Table), so they do not implicate specific pathways that might be involved in cooperative predation. This observation could be due to the small number of genes we examined, which is reminiscent of the broad spectrum of genes identified as modifiers of cooperative multicellular development in *D*. *discoideum* in larger genetic screens [9, 11]. It is therefore possible that cooperation in general is a rapidly evolving trait that might involve multiple pathways. We also note that none of the mutants was completely unable to grow on Gram(–) bacteria and that all of the mutants grew like the wild-type at high cell densities, further supporting the idea that multiple pathways are involved.

The definition of cooperation implies the production of public goods [5]. We found that *D*. *discoideum* secretes stable soluble factors that mediate cooperation and the synergy results suggest the involvement of secreted goods. These experiments also showed that not all pairs of mutants synergize and not all mutants benefit from the presence of wild-type secretions. These findings argue against the possibility of cross-feeding, in which the amoebae might leak out enough nutrients to support the growth of neighboring cells. The observation that *D*. *discoideum* secretes factors that mediate the degradation of bacterial biofilms supports and extends these conclusions [43]. We also found evidence for cheating during cooperative predation, which supports our conclusion since cheating is common in cooperative biological systems. We only found one instance of cheating behavior among the seven strains we examined, so it is unclear how pervasive this behavior might be. However, it should be noted that the first obligatory cheater mutation ever characterized in *D*. *discoideum* was also found in only one mutant strain out of a genetic screen of several thousand mutants [44]. Subsequent studies identified numerous facultative cheaters, suggesting that cheating in general is quite pervasive [9, 10]. The cheating ratio we observed here for predation was 60:40 after 8 hours of incubation, whereas the starting ratio was 50:50, which was the average ratio found in facultative developmental cheaters [9]. Therefore, although the evidence is not overwhelming, we conclude that cheating is a plausible component of cooperative predation in *D*. *discoideum*. Kin-recognition is known to protect against cheating during development [45] and it would be interesting to examine if it is involved in cooperative predation as well.

Previous studies have shown that *D*. *discoideum* kill bacteria by phagocytosis and that the amoebae engulf the bacteria before they kill them [7, 46]. The mechanisms utilized by the amoebae are highly conserved in mammalian immune cells, which makes *Dictyostelium* an excellent model system for studying the cell-autonomous aspects of defense against bacteria [20]. Phagocytosis is the major means by which *D*. *discoideum* feeds on bacteria when the proportion of bacteria to amoebae is low, similar to the interaction between mammalian immune cells and bacteria in which phagocytosis is used as a weapon in the battle against bacteria. But *Dictyostelium* cells also encounter very dense populations of bacteria when their spores germinate in the presence of a growing bacterial culture, so phagocytosis might not be sufficient to combat bacteria under these conditions. Indeed, a recent study showed that *D*. *discoideum* amoebae kill the Gram(–) bacteria *K*. *pneumoniae* extracellularly at the edge of a growing plaque, where the density of bacteria is high [22]. That study also showed that the amoebae secrete antibacterial proteins that kill the bacteria in a cell-free system, as well as discoidin I, a lectin that protects the bacteria from predation. The extracellular antibacterial proteins could be related to the common goods we found in the current study, whereas the protection of bacteria against killing is related to the farming ability of *D*. *discoideum* [47]. Another extracellular mechanism for killing bacteria was demonstrated in developing *D*. *discoideum* S-cells, which secrete extracellular DNA nets that trap and kill bacteria [48]. It is also possible that the amoebae may be able to modify the bacterial cell surface, rather than kill the bacteria, thus adding a non-cell-autonomous factor that could facilitate the cell-autonomous process of phagocytosis. The cooperation between the amoebae that we describe here is an additional intriguing level in an already complex relationship between *Dictyostelium* and bacteria.

Cooperative predation has been the subject of numerous studies and theory in the field is supported by ample empirical evidence, but it has been difficult to test some of the theories by directed experiments [1]. The discovery of cheating during multicellular development in *D*. *discoideum* [10] and the finding that cheating is genetically tractable [44] established *D*. *discoideum* as a model organism for the study of cooperation and social evolution. Our current findings introduce this genetically tractable eukaryote as a model organism for the study of cooperative predation and predator-prey relationships. The experimental methods we used do not mimic the natural ecology of *D*. *discoideum*, so our conclusions only indicate the possibility of cooperative predation in nature. We also tested only laboratory strains that differ by single identifiable mutations, as opposed to the many genetic differences found between dueling strains in nature. Nevertheless, laboratory findings about cooperation during development are consistent with analyses of naturally occurring strains [16, 49], so it is quite possible that cooperative predation occurs in nature as well. The ecological significance of cooperative predation by *Dictyostelium* in the soil environment has yet to be defined.

## Supporting information

S1 Fig Additional density-dependence growth curves. We incubated *D*. *discoideum* cells in submerged culture in association with *K*. *pneumoniae* at the following densities: cyan– 1x10^4^ cells/mL, purple– 5x10^4^ cells/mL, and red– 1x10^5^ cells/mL. The graphs show the calculated cell density (y-axis) as a function of time (x-axis, hours). Solid lines represent the wild-type and dashed lines represent the mutant, as indicated inside each panel. Each point represents the mean of three independent replications and the bars are the standard error of the mean. * P ≤ 0.05 for 5x10^4^ cells/mL, + *P* ≤ 0.05 for 1x10^4^ cells/mL; Independent samples t-test for pair-wise comparisons between the wild type and the mutant at each time-point. The wild-type controls for the following mutants are identical: 1) MR01 and MR03, 2) MR04 and MR05.
(TIF)

S2 Fig Mutant strain growth on heat-killed *K*. *pneumoniae*. We incubated pure populations of *D*. *discoideum* wild-type and mutant cells at 5x10^4^ cells/mL (solid lines) and at 1x10^4^ cells/mL (dashed lines) in submerged cultures with heat-killed *K*. *pneumoniae* and counted the cells at the indicated times. The graphs show the calculated cell density (y-axis) as a function of time (x-axis, hours). Each experiment was performed in three independent replicates; the results are reported as the mean and the bars represent the standard error of the mean. In all the graphs, the wild type is represented in blue and the other colors represent different mutants. (a) Yellow–MR02, red–MR05, and purple–MR06. (b) Red–*cadA*^*−*^and yellow–MR08. (c) Red–MR01, and purple–MR04. (d) Yellow–*tirA*^*−*^and red–MR03. * P ≤ 0.05 for *tirA*^*–*^, + *P* ≤ 0.05 for MR03; One-way ANOVA and post-hoc Tukey’s HSD test for pair-wise comparisons between the wild type and the mutant at each time-point.
(TIF)

S3 Fig Mutant strain growth on *S*. *aureus*. We incubated pure populations of *D*. *discoideum* wild-type and mutant cells at 5x10^4^ cells/mL in submerged cultures with *S*. *aureus* bacteria and counted the cells at the indicated times. The graphs show the calculated cell density (y-axis) as a function of time (x-axis, hours). Each experiment was performed in three independent replicates. In all the graphs, blue represents the wild type and the other colors represent different mutants. The differences between the growth rates of the wild type in the three images represent the variability of growth on *S*. *aureus*. (a) Red: MR06, purple: MR04, cyan: *cadA*^*–*^. (b) Red: MR08, yellow: MR02, purple: MR01. * P ≤ 0.05 for MR02, + *P* ≤ 0.05 for MR01 (c) Red: MR03, yellow: MR05, purple: *tirA*^*–*^. * P ≤ 0.05 for *tirA*^*–*^, + *P* ≤ 0.05 for MR05; # *P* ≤ 0.05 for MR03; One-way ANOVA and post-hoc Tukey’s HSD test for pair-wise comparisons between the wild type and the mutant at each time-point.
(TIF)

S4 Fig Soluble factors are involved in cooperative growth. We incubated pure populations of wild-type and mutant cells at 2.5x10^4^ cells/mL in submerged cultures in association with *K*. *pneumoniae* and placed mutant cells at the bottoms of two cell culture wells. We placed inserts in these wells, in which submerged cultures of amoebae and bacteria were deposited on a 0.4 μm membrane–one insert with matching mutant cells and one with wild-type cells. We counted the cell density (y-axis) at the indicated times (hours, x-axis). In the stacked bars, cell density in the insert is indicated in black and cell density in the well is in blue. Strain identity is indicated below each stacked bar; AX4:MR01 indicates that AX4 was in the insert and MR01 was in the well. Each experiment represents three independent replicates. The stacked bars represent the respective means and the error bars represent the standard error of the mean. Black asterisk: *P* ≤ 0.05 for the insert of wild type with wild type compared to the insert of mutant with mutant; blue asterisk: *P* ≤ 0.05 for the well of wild type with wild type compared to the well of mutant with mutant; #: *P* ≤ 0.05 for the well of the mutant with wild type compared to the well of the mutant with mutant; ns: not significant; One-way ANOVA and post-hoc Tukey’s HSD test.
(TIF)

S1 Table Strains used in this work. (DOCX)

S2 Table The area under the curve for wild type, mutant mix, and the z-score for each mutant mix presented in the synergy matrix. (DOCX)
