# Supplementary figures and images for: Cooperative predation in the social amoebae Dictyostelium discoideum
Source: PLoS One. 2019 Jan 9;14(1):e0209438. doi: 10.1371/journal.pone.0209438 (PMC6326426; doi:10.1371/journal.pone.0209438)

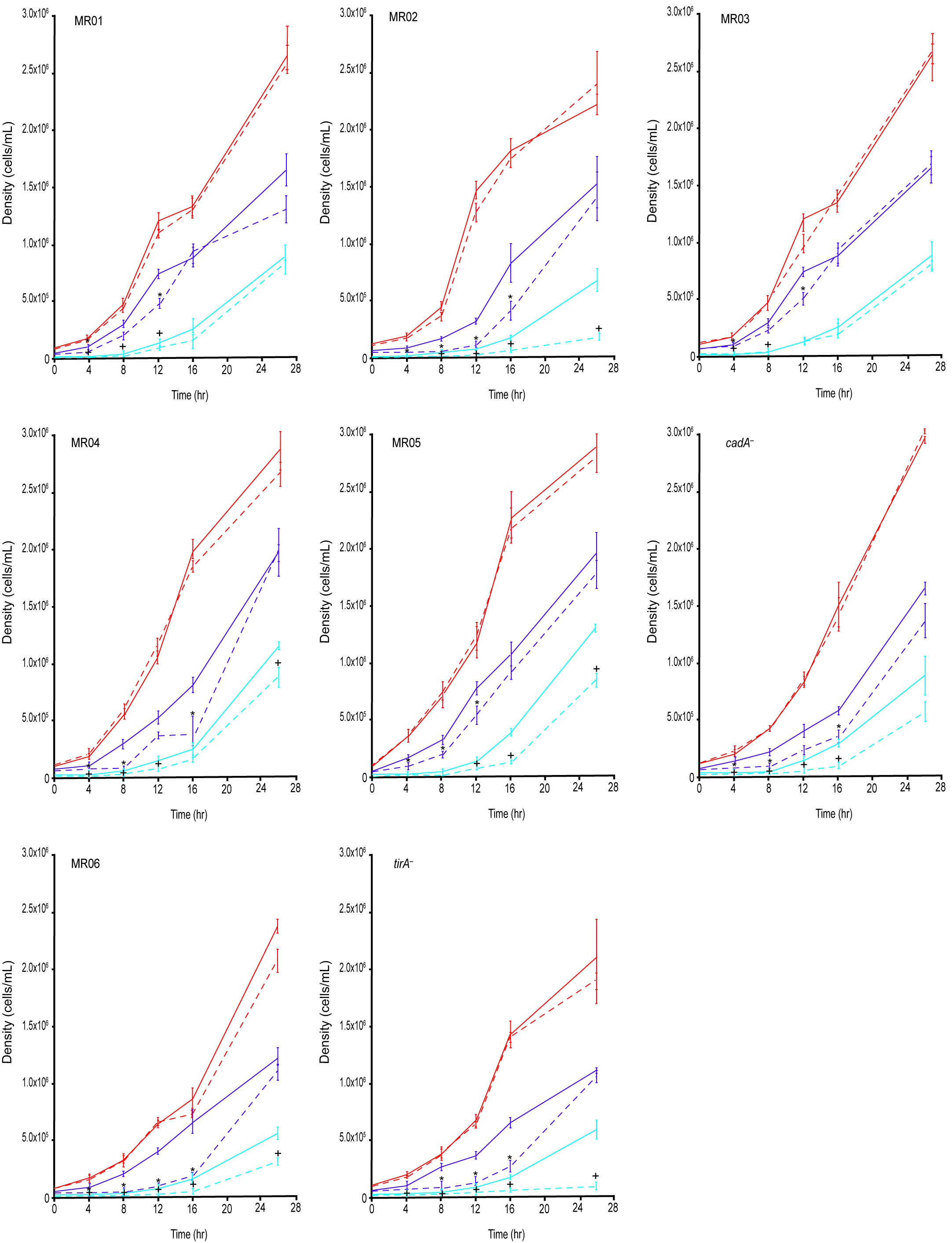

Supplement: S1 Fig — We incubated D. discoideum cells in submerged culture in association with K. pneumoniae at the following densities: cyan– 1x10^4^ cells/mL, purple– 5x10^4^ cells/mL, and red– 1x10^5^ cells/mL. The graphs show the calculated cell density (y-axis) as a function of time (x-axis, hours). Solid lines represent the wild-type and dashed lines represent the mutant, as indicated inside each panel. Each point represents the mean of three independent replications and the bars are the standard error of the mean. * P ≤ 0.05 for 5x10^4^ cells/mL, + P ≤ 0.05 for 1x10^4^ cells/mL; Independent samples t-test for pair-wise comparisons between the wild type and the mutant at each time-point. The wild-type controls for the following mutants are identical: 1) MR01 and MR03, 2) MR04 and MR05. (TIF) [file pone.0209438.s001.tif]

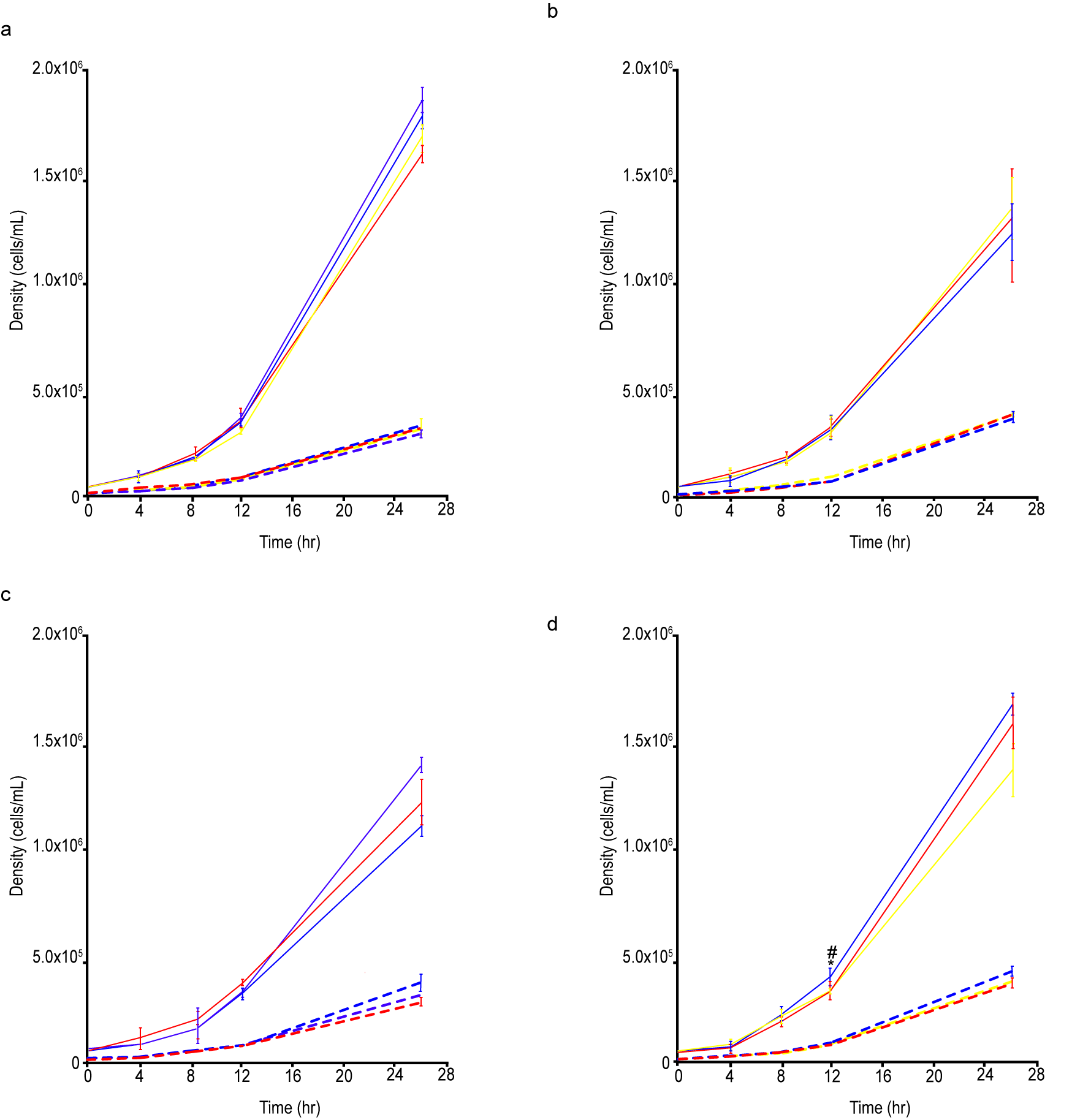

Supplement: S2 Fig — We incubated pure populations of D. discoideum wild-type and mutant cells at 5x10^4^ cells/mL (solid lines) and at 1x10^4^ cells/mL (dashed lines) in submerged cultures with heat-killed K. pneumoniae and counted the cells at the indicated times. The graphs show the calculated cell density (y-axis) as a function of time (x-axis, hours). Each experiment was performed in three independent replicates; the results are reported as the mean and the bars represent the standard error of the mean. In all the graphs, the wild type is represented in blue and the other colors represent different mutants. (a) Yellow–MR02, red–MR05, and purple–MR06. (b) Red–cadA−and yellow–MR08. (c) Red–MR01, and purple–MR04. (d) Yellow–tirA−and red–MR03. * P ≤ 0.05 for tirA–, + P ≤ 0.05 for MR03; One-way ANOVA and post-hoc Tukey’s HSD test for pair-wise comparisons between the wild type and the mutant at each time-point. (TIF) [file pone.0209438.s002.tif]

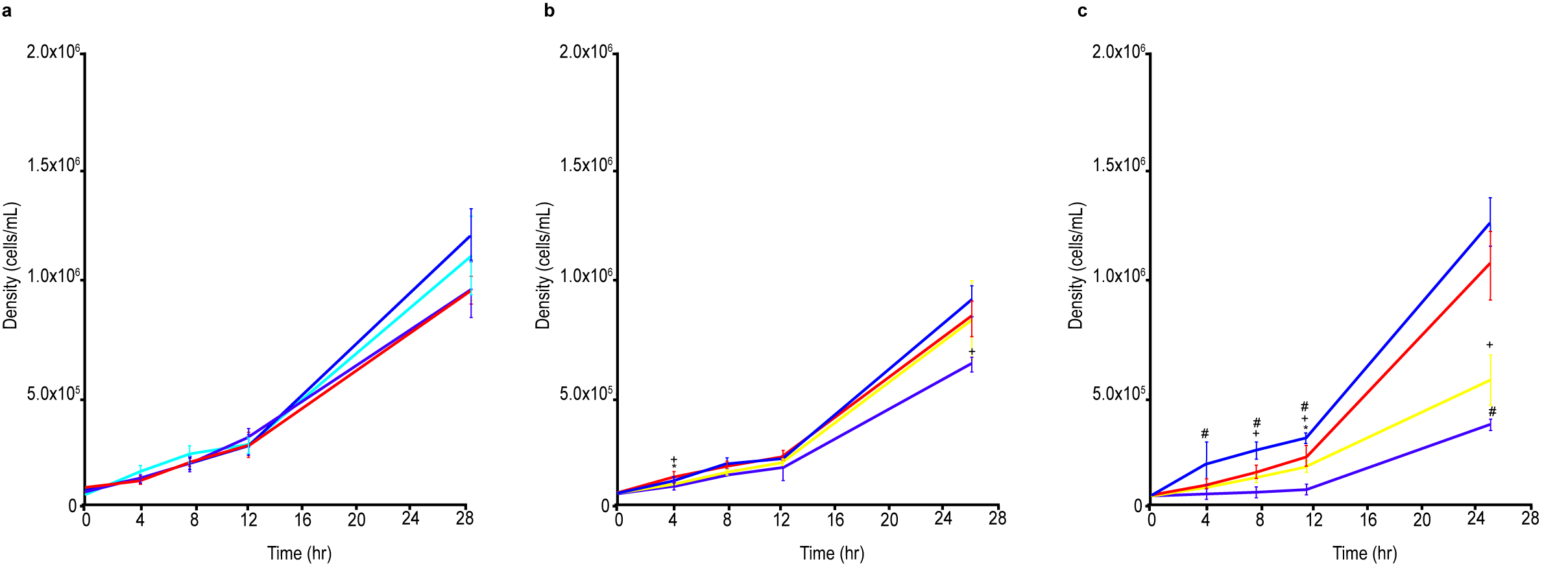

Supplement: S3 Fig — We incubated pure populations of D. discoideum wild-type and mutant cells at 5x10^4^ cells/mL in submerged cultures with S. aureus bacteria and counted the cells at the indicated times. The graphs show the calculated cell density (y-axis) as a function of time (x-axis, hours). Each experiment was performed in three independent replicates. In all the graphs, blue represents the wild type and the other colors represent different mutants. The differences between the growth rates of the wild type in the three images represent the variability of growth on S. aureus. (a) Red: MR06, purple: MR04, cyan: cadA–. (b) Red: MR08, yellow: MR02, purple: MR01. * P ≤ 0.05 for MR02, + P ≤ 0.05 for MR01 (c) Red: MR03, yellow: MR05, purple: tirA–. * P ≤ 0.05 for tirA–, + P ≤ 0.05 for MR05; # P ≤ 0.05 for MR03; One-way ANOVA and post-hoc Tukey’s HSD test for pair-wise comparisons between the wild type and the mutant at each time-point. (TIF) [file pone.0209438.s003.tif]

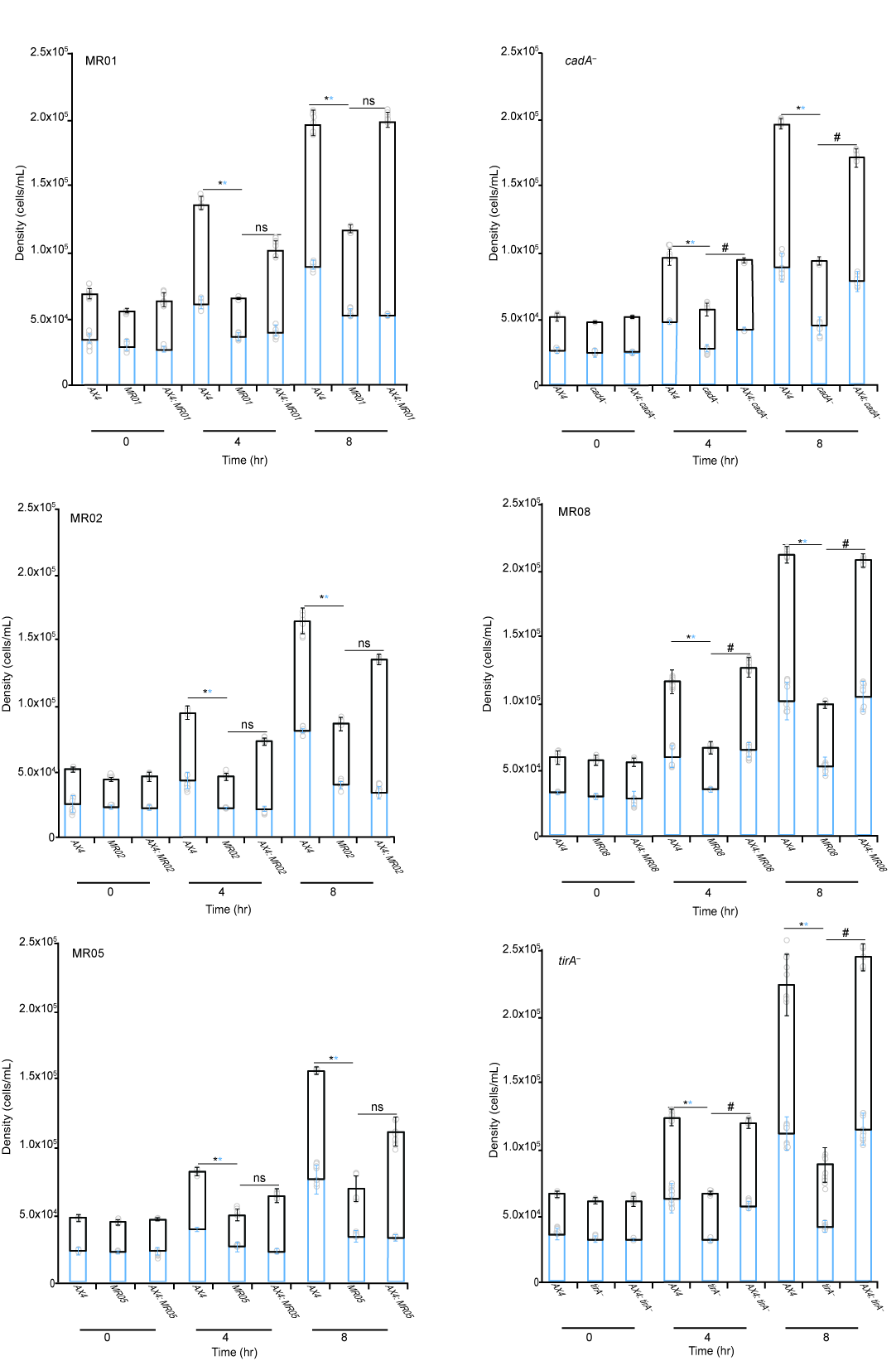

Supplement: S4 Fig — We incubated pure populations of wild-type and mutant cells at 2.5x10^4^ cells/mL in submerged cultures in association with K. pneumoniae and placed mutant cells at the bottoms of two cell culture wells. We placed inserts in these wells, in which submerged cultures of amoebae and bacteria were deposited on a 0.4 μm membrane–one insert with matching mutant cells and one with wild-type cells. We counted the cell density (y-axis) at the indicated times (hours, x-axis). In the stacked bars, cell density in the insert is indicated in black and cell density in the well is in blue. Strain identity is indicated below each stacked bar; AX4:MR01 indicates that AX4 was in the insert and MR01 was in the well. Each experiment represents three independent replicates. The stacked bars represent the respective means and the error bars represent the standard error of the mean. Black asterisk: P ≤ 0.05 for the insert of wild type with wild type compared to the insert of mutant with mutant; blue asterisk: P ≤ 0.05 for the well of wild type with wild type compared to the well of mutant with mutant; #: P ≤ 0.05 for the well of the mutant with wild type compared to the well of the mutant with mutant; ns: not significant; One-way ANOVA and post-hoc Tukey’s HSD test. (TIF) [file pone.0209438.s004.tif]
