# Supplementary material for: Cooperative predation in the social amoebae Dictyostelium discoideum
Source: PLoS One. 2019 Jan 9;14(1):e0209438. doi: 10.1371/journal.pone.0209438 (PMC6326426; doi:10.1371/journal.pone.0209438)
Supplement: S1 Table — (DOCX) [file pone.0209438.s005.docx]

**S1 Table. Strains used in this work**

| **Strain** | **Description ^a^** | **Insertion / deletion ^b^** | **Neighboring genes ^c^** | **Gene annotation** | **Reference / Acc. number** | **comments** |
| --- | --- | --- | --- | --- | --- | --- |
| AX4 | Parental strain |  |  |  | (*24*) |  |
| MR01 | REMI strain | chr3:2,744,347 | 374 bp upstream of *DDB_G0279951* and 1,053 bp upstream of *tRNA-Val-AAC-13* | *DDB_G0279951*: highly conserved protein containing an alkyl hydroperoxide reductase/thiol specific antioxidant/mal allergen domain  *tRNA-Val-AAC-13:* valine tRNA | This work  (SRR7765341) |  |
| MR02 | REMI strain | chr3:2,609,227 | 304 bp upstream of *lsm1* and 521 bp upstream of *DDB_G0306976* | *lsm1*: Like-SM protein: known to function in mRNA splicing and mRNA degradation  *DDB_G0306976*: unknown | This work  (SRR7765342) |  |
| MR03 | REMI strain | chr1:165,781  chr1:3,574,840  chr3:2,689,053  (chr3F:2,860) | In *DDB_G0267356*  In *DDB_G0270954*  In *DDB_G0305714*  In *DDB_G0294320* | *DDB_G0267356, DDB_G0270954* and *DDB_G0294320:* long terminal repeat retrotransposon Skipper GAG-PRO-POL  *DDB_G0305714:* Skipper ORF2 | This work  (SRR7765343, SRR7765344) | Inconclusive mapping due to insertion in a repetitive element |
| MR04 | REMI strain | chr5:826,186 | 540 bp upstream of *mkcC* and 452 bp downstream of *DDB_G0287851* | *mkcC*: serine/threonine kinase  *DDB_G0287851:* homolog of human insulin-degrading enzyme | This work  (SRR7765345) | Inconclusive mapping due to complex insertion(s) |
|  | REMI strain | chr5:957,464 | In *DDB_G0288003*  401 bp from the 3’ end. | *DDB_G0288003*: EGF-like domain-containing protein; putative N-terminal signal sequence, C-terminal anchoring transmembrane domain and one EGF-like domain putative protein |  |  |
| MR05 | REMI strain | chr3:4,896,570 | 1,866 bp upstream of *DDB_G0281735* and 889 bp downstream of *DDB_G0281737* | *DDB_G0281735:* Putative transmembrane protein  *DDB_G0281737*: Inositol polyphosphate multikinase | This work  (SRR7765346) |  |
| MR06 | REMI strain | chr2:6,197,246 | 9 bp upstream of *DDB_G0276015* | *DDB_G0276015*: Leucine-zipper-like transcriptional regulator 1 | This work  (SRR7765347) | This insertion is accompanied with a deletion of 89 bp of the 5’ end of *DDB_G0276015* |
| MR08 | REMI strain | chr5:4,726,657 | 2,362 bp upstream of *gefK* and 1,026 bp upstream of *gacL* | *gefK*: Ras guanine nucleotide exchange factor  *gacL*: RhoGAP domain-containing protein | This work  (SRR7765348) |  |
| *cadA^–^* | Inactivation of CadA | Chr4:3,656,555 | In *cadA* | *cadA*: Calcium dependent cell adhesion molecule-1 | This work | The CadA protein is not detectable by Western blot. |
| *tirA^-^* | Deletion Mutant of TirA | Amino acids 815-965 | TIR-domain replaced with a pGEM3-derived plasmid containing a BSR cassette and a GFP coding sequence | *tirA*: Toll-interleukin receptor domain-containing protein | (*39*) |  |

a – all the strains, except for AX4 exhibit defective growth on Gram(–) bacteria.

b – Insertion sites (nucleotides) were mapped to the *D. discoideum* genome on dictyBase, May 2009.

c – For insertions outside the coding region of a gene, we report the closest genes within 1.1 kb of the insertion site.
