# Supplementary material for: Cooperative predation in the social amoebae Dictyostelium discoideum
Source: PLoS One. 2019 Jan 9;14(1):e0209438. doi: 10.1371/journal.pone.0209438 (PMC6326426; doi:10.1371/journal.pone.0209438)
Supplement: S2 Table — (DOCX) [file pone.0209438.s006.docx]

**S2 Table. Quantitative data from synergy experiments**

| **Mutant Mix** | **Area under the curve - WT** | **Area under the curve - mix** | **AUC mix – AUC WT** | **Z-score** |
| --- | --- | --- | --- | --- |
| MR01+MR02 | 1868333 | 1040833 | -827500 | -0.70 |
| MR01+MR03 | 1986667 | 717500 | -1269167 | -1.34 |
| MR01+MR04 | 3209167 | 803333 | -2405833 | -2.97 |
| MR01+MR05 | 3075000 | 2875000 | -200000 | 0.20 |
| MR01+cadA^–^ | 2341667 | 2804167 | 462500 | 1.15 |
| MR01+MR06 | 1585000 | 1271667 | -313333 | 0.03 |
| MR01+MR08 | 1666667 | 1408333 | -258333 | 0.11 |
| MR01+tirA^–^ | 1763333 | 1696667 | -66667 | 0.39 |
| MR02+MR03 | 2718542 | 2590833 | -127708 | 0.30 |
| MR02+MR04 | 1770833 | 1877500 | 106667 | 0.64 |
| MR02+MR05 | 4972500 | 2991667 | -1980833 | 0.21 |
| MR02+cadA^–^ | 1912500 | 1721667 | -190833 | 0.21 |
| MR02+MR06 | 1874167 | 1965000 | 90833 | 0.61 |
| MR02+MR08 | 2064167 | 1665833 | -398333 | 0.63 |
| MR02+tirA^–^ | 1697500 | 1803333 | 1803333 | 0.64 |
| MR03+MR04 | 1834167 | 2008333 | 174167 | 0.73 |
| MR03+MR05 | 2497500 | 620000 | -1877500 | -2.21 |
| MR03+cadA^–^ | 2341667 | 2440000 | 98333 | 0.62 |
| MR03+MR06 | 2064167 | 1857500 | -206667 | 0.19 |
| MR03+MR08 | 2064167 | 2096667 | 32500 | 0.53 |
| MR03+tirA^–^ | 1697500 | 1648333 | -49167 | 0.41 |
| MR04+MR05 | 3350833 | 4147500 | 796667 | 1.63 |
| MR04+cadA^–^ | 1834167 | 1552500 | -281667 | 0.08 |
| MR04+MR06 | 2450000 | 3009167 | 559167 | 1.29 |
| MR04+MR08 | 3209167 | 1318333 | -1890833 | -2.23 |
| MR04+tirA^–^ | 2063333 | 2008333 | -55000 | 0.40 |
| MR05+cadA^–^ | 1862500 | 1747500 | -115000 | 0.32 |
| MR05+MR06 | 2468333 | 2353333 | -115000 | 0.32 |
| MR05+MR08 | 1862500 | 654167 | -1208333 | -1.25 |
| MR05+tirA^–^ | 1763333 | 1721667 | -41667 | 0.54 |
| cadA^–^+MR06 | 1585000 | 570833 | -1014167 | -0.97 |
| cadA^–^+MR08 | 1770833 | 648333 | -1122500 | -2.10 |
| cadA^–^+tirA^–^ | 1761667 | 1998333 | 236667 | 0.82 |
| MR06+MR08 | 2064167 | 960000 | -1104167 | -1.10 |
| MR06+tirA^–^ | 2580000 | 2732500 | 152500 | 0.70 |
| MR08+tirA^–^ | 2063333 | 1779167 | -284167 | 0.08 |
